# Exploring the Implementation of a New Nursing Role Using the CFIR–ERIC Approach: A Qualitative Study

**DOI:** 10.1155/jonm/8513187

**Published:** 2026-05-16

**Authors:** Marianne Saragosa, Paula Shing, Zahra A. Hirji, Kara Ronald, Lianne Jeffs

**Affiliations:** ^1^ Science of Care Institute, Toronto, Canada; ^2^ Lunenfeld-Tanenbaum Research Institute, Toronto, Canada, lunenfeld.ca; ^3^ University of Toronto Institute of Health Policy Management and Evaluation, Toronto, Canada; ^4^ Sinai Health, Toronto, Canada; ^5^ University of Toronto Department of Physical Therapy, Toronto, Canada

## Abstract

**Aim:**

This study aimed to retrospectively describe the implementation barriers and facilitators when introducing a new nursing role (RPN) using the Consolidated Framework for Implementation Research (CFIR) and to map implementation strategies onto the Expert Recommendations for Implementing Change (ERIC) factors to identify relevant strategies supporting new role implementation in the acute care setting.

**Design:**

A qualitative descriptive study embedded in a multiphase implementation evaluation study.

**Methods:**

Semistructured interviews with organizational leaders, implementation leaders, and nurses, combined with document analysis, were analyzed using the CFIR deductively to identify barriers and enablers mapped to the ERIC tool for strategies guiding implementation.

**Results:**

Participants (*N* = 25) represented organizational leaders (*n* = 3), implementation leaders (*n* = 11), RNs (*n* = 4), and RPNs (*n* = 7) from October 2024 to January 2025. Seventeen CFIR constructs across five domains were identified. Common themes of enablers include factors in the inner setting, such as a tension for change, mission alignment, and innovation compatibility. Many enablers were also found in the implementation process, which involved teaming, assessing context and needs, engaging, tailoring strategies, and reflecting and evaluating. Barriers to implementation were found in the inner setting, including culture and limited access to knowledge and information about the role. Eleven implementation strategies were identified to address or amplify the barriers or enablers, including involving and preparing staff for the introduction, developing education material, and identifying champions.

**Conclusion:**

The introduction of the RPN role in acute care was shaped by organizational readiness, leadership support, and workforce needs, while challenges related to role clarity and team integration remained. Implementation science approaches may help guide strategies to support the successful integration of new nursing roles within hospital care delivery models.

**Reporting Method:**

This study adhered to COREQ guidelines.

**Patient or Public Contribution:**

No patient or public contribution.

## 1. Introduction

Nurses are the largest proportion of healthcare providers globally, with 450,000 nurses working in Canada [1]. Long‐standing issues, including economic pressures, excessive job demand, dissatisfaction, and burnout—amplified by the COVID‐19 pandemic [2]—have contributed to an 85.8% increase in nursing job vacancies in 2021 [3] and to the evolution of the healthcare workforce into new roles and skill mixes [4]. In many countries, this has led to the development and increase of other categories of nurses (e.g., registered practical nurses [RPNs] in Canada). In Canada, the number of registered nurses (RNs) providing direct hospital care decreased by 1% in 2022, while RPNs increased by 3% [5]. Over 27% of RPNs were employed in Ontario’s [6] and 47% in Canada’s acute care hospitals [7]. With the increased demand for nursing care amid shortages [8, 9], hospitals increasingly employ RN/RPN skill mix models to ensure quality patient care and outcomes [10]. While RNs and RPNs are expected to apply their knowledge, skills, and judgment to practice safely and adapt to different care settings, their entry‐to‐practice requirements differ [11]. For RNs, an applicant must have a baccalaureate nursing degree, whereas an RPN requires a practical nursing diploma [12]. There is a small but growing evidence base on the RN/RPN skill mix model as a mitigating strategy to staffing issues [13] and its impact on collaborative practice [13], control over practice [13], and patient care quality [14].

Historically, challenges related to role differentiation, scope of practice and perceived hierarchy have been reported when introducing RPN roles in acute care settings [4]. These challenges can contribute to role conflict [15], role clarity [15, 16], and tensions within nursing teams [17]. For example, a recent study reported higher levels of role conflict among a random sample of Canadian RNs than among RPNs in medical and surgical units [18]. Role conflict and lack of clarity have been associated with burnout [19] and turnover [20] in nurses working in acute care. Previous efforts to optimize the RN/RPN skill mix model in Ontario have included the development of an RN/RPN utilization toolkit [21] and the WeRPN’s Strategic Plan [9]. A recent rapid review evaluating skill mix models of care found positive outcomes in nurse and patient satisfaction and in costs and outlined successful implementation strategies (e.g., timely education of all nursing staff regarding job descriptions and roles) [22]. However, despite the increasing use of RN/RPN skill mix models, limited research has examined how these models are introduced and operationalized within complex healthcare environments [23].

Introducing new models of care represents a significant organizational change that requires careful planning, engagement, and evaluation [4]. Implementation science offers a useful lens for considering contextual factors that often work for or against implementation efforts [24]. When applied to integrating new evidence into clinical practice, implementation science approaches have been associated with improved patient safety, better outcomes, and more efficient use of resources [25]. Despite these benefits, recent research indicates that implementation strategies and theoretical frameworks are frequently absent when introducing new nursing care delivery models [26]. Given the complexity of introducing a care delivery model while standard nursing care continues, an evidence‐informed implementation approach is warranted.

To address this gap, this study retrospectively described barriers and facilitators in implementing a new nursing role (RPN) using the Consolidated Framework for Implementation Research (CFIR) and mapped strategies onto the Expert Recommendations for Implementing Change (ERIC) factors to identify those that support new role implementation in acute care. Applying an implementation science lens, the study aimed to examine how the RN/RPN skill mix model was adopted and introduced in practice and to identify key barriers and enablers associated with its implementation.

## 2. Materials and Methods

### 2.1. Study Aim

This study aimed to retrospectively describe the implementation barriers and facilitators when introducing a new nursing role (RPN) using the CFIR and to map implementation strategies onto the ERIC factors to identify relevant strategies supporting new role implementation in the acute care setting.

### 2.2. Design

This qualitative study represents one component of a broader mixed‐methods evaluation examining the implementation and impact of the RN/RPN skill mix model at a large academic teaching hospital in Toronto, Canada. The qualitative component aimed to explore nurses’ and implementation leaders and facilitators’ experiences and perceptions of the implementation process, while other components of the evaluation include patients and care partners and the impact of the introduction on quantitative and organizational data sources. Findings across these components will be integrated in a subsequent publication to enable triangulation and provide a comprehensive understanding of the implementation and impact of the skill mix model. For the purpose of this study, a qualitative descriptive study design was used to achieve the study’s aim. Reporting was aligned with the Consolidated Criteria for Reporting Qualitative Research (COREQ) [27], which is reported in a Supporting Information ([28] (See Supporting File 1).

### 2.3. Conceptual Frameworks Underpinning the Study

An integrated implementation science approach was used to inform the evaluation with a focus on determinants and contextual factors used to explain implementation outcomes that can be used to identify the best implementation strategies to address contextual determinants. Our study used the updated CFIR [24]. CFIR is a widely used framework from the implementation science literature consisting of five domains and 48 related constructs representing the entirety of the implementation process [29]. The domains include characteristics of the intervention (in this case, implementation of the RPN role), the inner setting (the hospital), the outer setting (the context in which the hospital exists), individual characteristics of the implementers (hospital staff), and the processes used for introducing the new role. As a “determinants framework,” CFIR is meant to be applied deductively to identify barriers and enablers that influence the targeted implementation outcomes [30].

Additionally, the ERIC [28] taxonomy was used to categorize implementation strategies that supported the role introduction. The ERIC taxonomy comprises 73 discrete implementation strategies clustered into nine clusters [31]. The ERIC framework provides specific implementation strategies, their definitions, and descriptions of their applications, which are associated with the CFIR domains: innovation, outer setting, inner setting, individuals, and implementation process [24]. To allow for capacity to develop robust implementation plans using ERIC strategies associated with specific CFIR determinants, the CFIR–ERIC matching tool was developed [32]. The tool, developed by experts in implementation science, had respondents endorse the strategies. Strategies endorsed by ≥ 50% of the experts were considered “Level I” strategies, and strategies supported by 20%–49.9% were deemed as “Level 2” strategies [32]. The CFIR–ERIC matching tool has been used widely to generate outputs of barriers and facilitations matched to CFIR determinants to ERIC strategies [33, 34]. These strategies could help identify effective implementation strategies for the future scale of new models of care into practice. The CIFR and ERIC domains are provided with corresponding short descriptions in a Supporting Information (Supporting File 2) [24, 28].

Before implementation, hospital leadership and nursing management undertook several preparatory activities to facilitate the integration of the RPN role into the existing care delivery model starting in 2022. These activities included workforce planning, role clarification, and operational adjustments within clinical units. While the CFIR framework guided the analytical approach used in this study to explore implementation determinants, the introduction of the RPN role was part of an organizational workforce strategy rather than a formally structured implementation model. Instead, CFIR was used to guide the analysis of implementation determinants influencing the introduction of the RPN role. Interview transcripts and relevant documents were coded using CFIR constructs to identify barriers and facilitators across implementation domains. Following identification of these determinants, the implementation strategies described in the ERIC compilation were used to retrospectively map the strategies employed during the role introduction and to assess their alignment with the identified implementation determinants.

### 2.4. Setting

This study was conducted at a 442‐bed academic teaching hospital in Toronto, Canada. In July 2024, the hospital introduced the RPN role on three inpatient units: labor and delivery, postpartum, and the main operating room (OR); however, the preimplementation process began in 2022 with the launch of an environmental scan and later the professional care delivery model (PCDM) governance structure, which established an implementation plan in 2023 and that met various milestones. A phased approach was used to gradually introduce the RPN role, with the expectation that the role would be rolled out in other departments as appropriate.

### 2.5. Participants and Recruitment

Participants were purposively recruited from clinical areas where the RPN role has been introduced as part of the first phase of the new skill mix model, including the main OR, postpartum (i.e., “Mother Baby Unit”), and labor and delivery. A sampling matrix was created to account for the experiences and perspectives of those involved in the implementation at the unit and organizational levels. This matrix guided the recruitment of three different participant groups: organizational leaders/facilitators (e.g., senior executives, directors) within the organization, but not directly involved in delivering the intervention; implementation leaders responsible for implementing the new skill mix (e.g., nurse educators, and patient care managers); and nursing staff involved in providing care (e.g., RPNs and RNs). The inclusion criteria were that participants had been identified as relevant to or engaged in introducing the RPN role.

For recruitment, the principal investigator invited potential participants through an invitational email sent via existing listservs with the consent form. A reminder email was sent approximately 2 weeks after the initial invite. Those interested in participating contacted the PI or the research coordinator. All participants provided written consent before any data were collected. Following the first round of interviews, snowball sampling [35] was used to identify additional participants, using suggestions from participants as to other individuals who might provide different perspectives on implementing the new role.

### 2.6. Data Collection

Qualitative data were collected through individual semistructured interviews from October 2024 to March 2025. Most interviews were conducted virtually using a hospital‐approved Zoom account, while a select few occurred over the phone. The semistructured interview guides were developed by the research team based on the study objectives and informed by constructs from the CFIR framework. Questions were organized around key CFIR domains, and separate interview guides were developed for three participant groups: organizational leaders, implementation leaders (e.g., nurse educators and patient care managers), and nursing staff (e.g., RNs and RPNs), in order to capture perspectives across different roles involved in the implementation. During interviews, follow‐up probes were used to encourage participants to elaborate on their experiences and perspectives. Minor refinements to question wording were made during the study to improve clarity while maintaining consistency with the overall guide (see Supporting File 3). Two research team members (MS, LZ), trained in qualitative interviewing and implementation science, conducted all the interviews. All interviews were recorded and transcribed verbatim. After the interview, the participant received a $50.00 electronic gift card from their preferred retailer.

In addition to interview data, implementation meeting PowerPoints and minutes, an implementation toolkit (e.g., question logs, checklists, and guidelines), training and education (e.g., new hire orientation), organizational communication (i.e., memos), and orientation evaluation survey data were also gathered as part of the document analysis.

### 2.7. Data Analysis

Directed content analysis was used as the qualitative analysis approach [36]. Audio recordings were transcribed, and two members (MS, MR) independently reviewed the transcripts to familiarize themselves with the data. The CFIR framework was used deductively to develop the categorization matrix, with the interview transcripts and documents serving as the units of analysis [36]. To enhance rigor and consistency of the coding process, two transcripts were independently coded by both analysts and then compared to identify discrepancies, using predefined codes based on the CFIR. Differences were discussed and resolved through consensus, and an initial codebook was developed based on CFIR constructs. The codebook was iteratively refined throughout the analysis. Following the identification of barriers and enablers, the implementation strategies that supported the introduction of the RPN role were extracted from the data and mapped using the CFIR–ERIC matching tool (available at https://cfirguide.org/choosing-strategies/) [32]. In the final stage, both interview transcripts and relevant organizational documents were coded deductively to identify evidence of ERIC strategies used during implementation. Discrepancies were resolved through discussion, and the coding framework was refined as needed. Data management and coding were supported using Microsoft Word.

To enhance the trustworthiness of findings, several strategies were employed. Credibility was supported through independent coding by two researchers with extensive qualitative research experience and regular discussions to resolve discrepancies and refine the codebook. Dependability was strengthened through the use of a structured analytical approach guided by the CFIR framework and ERIC matching tool. Confirmability was supported by maintaining an audit trail, including iterations of the codebook and analytic memos. Finally, integrating multiple data sources (interviews and documents) enabled triangulation and enhanced the depth and robustness of the findings.

### 2.8. Ethical Considerations

This study was conducted within a larger implementation evaluation study and was approved by the Mount Sinai Hospital Research Ethics Board (REB#24‐0127‐E) on August 23, 2024. As the study was conducted within the organization where the first author holds an embedded nurse scientist role, some study participants were known to the research team as professional colleagues. However, no direct supervisory or managerial relationship existed between the researchers and participants. All participants provided written consent and were informed during the consent process that they would receive a gift card as a token of appreciation following completion of the interview. They were also informed of the voluntary nature of their participation and the confidentiality of their responses before the interview to support an open dialog.

### 2.9. Reflexivity Statement

The core investigative team consisted of female researchers and hospital leaders from nursing, health disciplines (e.g., physiotherapy), and nonclinical disciplines with various educational qualifications, from postsecondary education to PhD preparation. The team also included a range of research experience, from applied qualitative methods to more senior scientists with advanced research competencies and expertise in using the CFIR framework in previous studies [37]. Reflexivity was integrated throughout the research to examine how the team’s backgrounds and perspectives might influence data interpretation. The team, comprising nurse scientists and organizational leaders experienced in nursing workforce research and implementation science, held regular discussions during analysis to reflect on how disciplinary views and assumptions affected coding. Analytic memos recorded reflections on themes, alternative views, and researcher influence. These reflexive discussions improved transparency and kept interpretations aligned with participants’ accounts.

## 3. Findings

### 3.1. Characteristics of Participants

A total of 25 interviews were conducted with the following participants: organizational leaders (*n* = 3), implementation leaders (*n* = 11), RNs (*n* = 4), and RPNs (*n* = 7). Interviews lasted between 22 and 77 min, with an average of 50 minutes. Since the introduction of the RPN in this organization could identify the participants, the data on participant characteristics were limited to their roles and employment history. In this context, most participants identified as implementation leaders (e.g., nurse manager and educators) (*n* = 11), followed by RPNs (*n* = 7). The duration within their stated role ranged from 3 months to 8 years, while their tenure with the organization spanned 3 months to 32 years. See Table 1 for participant characteristics.

**TABLE 1:** Participant characteristics.

Current role (*n*)	
Organizational leader (e.g., Directors)	3
Implementation leaders (e.g., managers, educators, etc.)	11
RPN	7
RN	4
Setting (*n*)	
Main OR	5
Labor and delivery	7
Mother Bay Unit	6
Corporate	7
Duration of experience in current role (*Y*)	
Range	0.25–8
Mean	3
Duration of experience with organization (*Y*)	
Range	0.25–32
Mean	10
Duration of nursing experience (*Y*)	
Range	0.5–32
Mean	11

### 3.2. Findings From Corresponding CFIR Domains

Seventeen (*n* = 17) constructions from five domains were described as either a barrier, enabler, or both.

#### 3.2.1. Outer Setting

Narrative emerged within the *outer setting* domain, focusing on “systemic conditions” and “external pressure.” The construct of systemic conditions included environmental factors related to the nursing shortage, which was worsened by the COVID‐19 pandemic. This external driver was cited consistently as a rationale for introducing RPNs. It was recognized that, since the pandemic, nurses have moved away from their roles or the profession altogether, as noted in the following quote:

*But since COVID, I think there has been a shift across all organizations, recognizing that many nurses who may have been around and in practice back then have either shifted away from nursing or gone to different areas. And so you needed to look at what else we could do to sustain a healthcare system that could provide care for people who still were not going away, despite nurses leaving*. *It was an opportunity to say that other qualified people could do much of this work, and you also started to see other organizations doing it*. (P015)

Consequently, an additional and related construct was external pressure from comparable organizations already adopting the RPN role and potentially improving the efficiency of staffing models, which was compounded by the local chronic nursing shortage. One participant mentioned key performance measurements about balancing the staffing budget while managing rising overtime, sick time, and turnover.

*We know that at other organizations, all of their postpartum patient care is provided by RPNs. So that′s already a known factor that the RNs know about as well; obviously, they have friends or families that work in other places, deliver in other places, so they know that also*. *You can look online and look at those job postings*. (*P001*)

#### 3.2.2. Inner Setting

Many of the *inner setting* domain constructs were identified, including the organizational “culture” that often overlapped with “access to knowledge and information” about introducing RPNs into the nursing skill mix [innovation]. Following this, “tension for change” and “mission alignment” influenced the innovation’s “compatibility .” Many participants referred to the hospital’s historical all‐RN workforce as a core aspect of the cultural ethos and climate in which the organization operates. For those with long‐standing careers at the organization, the staff were “proud to say that it was RN [model]” (P015) or that the organization had achieved Magnet status, *And also Magnet status was another thing that came up before that everyone thought that being a Magnet hospital, you only have an all-RN staff*’ (P018). Given the embeddedness of this organization within a culture that valued its RN workforce, exposure to the RPN role, including “access to knowledge and information,” was initially limited. This, in turn, acted as a barrier to the implementation, including resistance to change and negative perceptions of the role.

*Some RNs were entrenched in the hospital culture, where it was just that they were the ones who were having difficulty understanding what it would be like if I were alone with an RPN. What do I do, you know? And what if there′s something that happens in an emergency, you know, who′s going to come and help me? And all of that.* (P002)

However, the organizational culture was also described as inclusive, for example, “*We are very inclusive. We put a lot of emphasis on teamwork and diminishing those hierarchies that happen in healthcare*” (P008). Participants acknowledged receiving the knowledge and information about introducing RPNs into the nursing skill mix by researching, learning from the internal hospital leaders and other peer organizations, and referring to the nursing regulating body to understand more about the role.

*I did talk to my peer coordinators at different hospitals*. *I reviewed the College of Nurses, the guidelines for differentiating the RN and RPN roles, and the Registered Nurses’ Association of Ontario’s best-practice guideline*. (P010)

The innovation was also driven in parallel by an internal “tension for change” and an alignment with the broader “mission” of the organization, which was to deliver a PCDM. A tension was identified from organizational staffing concerns, including long‐standing nursing vacancies, overtime hours, sick calls, and related vacation denials, which resulted in an overburdened nursing workforce and turnover rates. Tension also occurred in response to the opening of new departments and challenges with finding RN nursing staffing. This tension for change was described in the following quote:

*We had huge vacancies, and the vacancy rate was so high. I don’t think anyone blinked; there was no way we were giving anyone’s position away. Like, at this point, it was like, we just got to fill these positions, like at this point we need warm bodies*… (P016)

The launch of the PCDM work, which aimed to ensure the right role cares for patients at the right time, catalyzed the implementation of the RPN role. The PCDM strategy enabled participants to imagine what the role could look like for patient services and provided a highly structured process for implementation.

*It′s important for us to look at the model of care and see that our role is to take care of the patients at the right time, and that they have the right supports in place. And so, it′s sort of an overall approach to make sure that we′re using our people to their full scope. (P005)*

Finally, the innovation’s compatibility was considered both an enabler and a barrier to the implementation process because of misunderstandings of the role and its perceived fit within existing clinical settings and workflows. For example, within the OR environment, the role seemed to be highly compatible as it fit well in the OR system as the “scrub nurse,” a role delineated and well‐accepted by other organizations, according to the following quote:

*I was first introduced to the OR RPN role at [hospital]. They were already entrenched in that operating room, and so they worked very symbiotically with the RNs. It quickly became known to me that the RPNs were just as much an asset to the OR as the RNs.* (P002)

Conversely, their compatibility with the organization was less clear on other clinical units, where perhaps the fit with the existing workflow was not as seamless when compared to the OR. For example, on the labor and delivery floor, the unpredictable nature of delivery limited the RPN’s role. This also may have called into question the role’s compatibility with that particular unit:

*It is a labour and delivery unit, so that′s predominantly what our patients come in for, and they [RPN] cannot take care of labouring patients with fetal health surveillance. And then, as well as any patients that are, I guess, not predictable…it limited the number of patients they can take.* (P006)

#### 3.2.3. Innovation Characteristics

Participants articulated the advantages and disadvantages of implementing the RPN role in the context of not implementing the role altogether. In this context, narrative was aligned with the following constructs: “relative advantage,” potential for adaptability, and “design” within the *innovation characteristics* domain. For example, participants reported that the implementation would address nursing shortages and “filling those gaps” [P018], improve the quality of patient care and efficiencies, and enhance staffing levels. For the latter, optimizing the nursing staffing model in the OR would reduce the workload and create capacity to perform more surgeries and even maintain the emergency OR room. This insight overlaps with reinforcing an “incentive system” within the *inner setting* noted in the following quote:

*It′s allowed us to deal with a backlog of surgeries that we couldn’t do during COVID. So you know, obviously, being able to do that because of this is very satisfying for stakeholders, not only the physicians and the other team members, but also our administration, because we have obligations around what we do with our funding models*. (P005)

However, a few perceived disadvantages to including RPNs in the nursing skill mix were also noted, some of which were driven by misconceptions of the role. For example, these disadvantages were centered on the ambiguity of their skill set, heavier patient assignments and RNs losing their jobs, for example,

*I think some of the nurses were worried that eventually RN positions won’t be filled with RNs and they’ll be replaced with RPNs, so over time, the dilution in the staffing pool will have more RPNs. Some of them have expressed worries that they will need to do more and that their workload will be heavier, given that they may need to take care of more critical patients compared to RPNs, so the patient assignment acuity is in question. There is also the ongoing worry that they are “cheaper labour*.*”* (P010)

The degree to which the role could be adapted or “grown” to meet the local needs of the organization was also often mentioned. In the OR, this was considered in the context of the RPN role, eventually expanding to a second circulating nurse. In other units, expansion included the RPN as the secondary nurses for delivery. The interviewed RPNs voiced their desire to adapt the role, given their experience with other organizations and their feelings that their scope of practice was not being utilized to its fullest.

*They don’t utilize us enough in the sense of…I think some of our skills are being wasted, or in the sense that they’re not allowing us to expand our role as yet to do more. Some of the L&D nurses may not know what our skill set is, and what we are allowed or not allowed, or can or cannot do, so they tend not to call on us when it’s like, “Hi, we’re here. We′re available*.*”* (P025)

The last construct of “design,” which speaks to how an innovation is presented, was for the RPN role about role clarity. Role clarity seemed quite evident in some cases and less so in others, depending on the context. Namely, the design of the role for the OR “aligned beautifully,” according to one participant; however, the design was compromised, perhaps when their responsibilities were not as clearly defined outside that of a scrub nurse. This lack of understanding seemed also to be reflected in the RPNs of their role. For example, one RPN participant said,

*The biggest struggle was figuring out my role and what I could do, because sometimes I would start doing something, and the RN would see me, saying, “Oh, are you…you can’t do that”. I’m like, “Oh, I didn’t know.” I’ve been doing the RN role this whole time, and so I just assumed that I could do fetal auscultation*. (P020)

#### 3.2.4. Implementation Process

The domain of the *implementation process* included the constructs of “teaming,” “assessing context,” “assessing needs,” “engaging,” “tailoring strategies,” and “reflecting and evaluating.” The first steps in the planning process for the implementation involved the intentional convening of key partners, coordinating, and collaborating on many different tasks. Therefore, from the beginning, it was mentioned that the leadership team helped create subcommittees of different working groups at different organizational levels. For example, one participant stated, “*There were a lot of implementation planning groups at various levels, like a corporate steering level, program level and then unit level planning happened*” [P005]. The intentional joining together helped to assess the implementation context and individual needs in the following ways before and during the implementation. For example, in partnership with unit‐based staff, leadership team members conducted validated, operational assessments across all the units to determine if RPNs can safely manage the patient care needs (acuity, complexity, and predictability). Some participants considered the “visibility of senior leaders” (P015) a facilitator. Collecting information on the needs of people also occurred in numerous ways, including requesting and answering questions and concerns from the direct care nurses, conducting staff focus groups and education assessments on the staff. One participant also mentioned the involvement of the “change management team,” which evaluated and supported the change readiness among the staff in the following way:

*At the beginning, this survey went out to all frontline staff to evaluate change readiness, and based on those results, it was able to inform unit leadership and change management on what needs to be done for this group of staff to help them through this transition*. (P003)

The implementation phase involved engagement and strategies tailored to the various assessments and teaming efforts. The *engaging* construct had the most significant data supporting evidence of engagement, and many considered these strategies to occur constantly, using different collaborative approaches and communication modalities (i.e., newsletters, question boxes, email, and huddles). Also noted in the interview and documents data is the engagement of the direct care nurses in various ways, from formal committees to seeking their input on guidelines and processes, and being preceptors or mentors to the incoming RPN cohorts, for example,

*We created a space on each unit for feedback, including any questions they wanted us to answer or address in our meetings, and then fed back the answers to them. We included any updates in our weekly huddle notes and huddled with each team every week*. (P024)

Relatedly, implementation strategies were often tailored to mitigate implementation barriers or to amplify enablers, which emerged from the engagement process, including tailoring specific tools or resources:

*So we’ve devised a comprehensive list of what an RN and an RPN can do that came out of our implementation committee*. (P002)

*We developed a new corporate tool, the patient assignment guidelines, and we went through numerous iterations because we wanted to ensure that we had the feedback from the Clinical Nurse Specialists, the Patient Care Managers, and Team Leaders to ensure that it made sense and that it applied to them*. (P003)

Other tools also involved competency lists, an orientation evaluation survey, an escalation tool, and a team leader retreat to gain practical experience with these tools, such as the patient assignment guidelines. Finally, the “reflecting and evaluation” construct included quantitative and qualitative feedback collected as part of the implementation process. Participants mentioned using a standardized evaluation of the orientation for newly hired RPNs. Another commonly referenced reflection and evaluation strategy was establishing communities of practice (CoPs). In these CoPs, RPNs met regularly with leadership team members to provide feedback on their experiences, which was used to address any concerns noted in the following quote:

*Having that weekly check-in made a big difference for us. The meetings let us decompress from the week and share what we experienced*. *Still, it was also just a safe space to discuss anything we had an issue with*. (P013)

Many nurses also reflected and evaluated in real‐time by giving informal feedback about their experiences or observations, including suggestions for improving unit orientation or specific practices. For example, one RPN participant described how feedback was provided and addressed for newer cohorts of RPNs seen in the following quote:

*So there were a few learning gaps, like we weren’t taught to check case parts at first, and that has been implemented now that there’s another cohort here. So, I think many gaps in our first cohort are now fixed because we mentioned things as they came up*. (P012)

#### 3.2.5. Characteristics of Individuals

Within the *individual* domain, participants shared their own “motivation” to implement the new role successfully, which was leveraged for some who had roles as implementation leaders to inform the implementation process. Motivation was informed by wanting the RPNs to be well‐received and not tolerating unprofessionalism; however, from an operations perspective, the successful implementation was also motivated by retaining nurses and improving patient flow and efficiencies.

*I’m known in the unit to really not have anybody not be nice to each other, and if there are any concerns, people feel very comfortable coming to me to help resolve those issues. So having the RPNs succeed in the unit on a human level was a goal of mine*. (P002)

### 3.3. Findings From CFIR–ERIC Mapping

Guided by the CFIR analysis, barriers, enablers, or both were identified and mapped using the ERIC matching tool. The CFIR–ERIC mapping tool identified 11 ERIC strategies (see Table 2) that received 45% or greater agreement, with most strategies falling in the inner setting, innovation characteristics, and implementation process. Evidence of the ERIC strategies used in practice supporting the implementation was also included. These examples included specific tools, such as an e‐module, quizzes, and an implementation toolkit, as well as considerations to eventually adapt the role to enhance the fit of the innovation with the context. It should be noted that some CFIR constructs do not have matched strategies in the matching tool, including systemic conditions, external pressure, structural characteristics, mission alignment, teaming, assessing context, and assessing needs.

**TABLE 2:** Level 1 and 2 strategies identified by the CFIR–ERIC matching tool.

CFIR domain	CFIR construct	CFIR construct enabler	CFIR construct barrier	Level 1 and 2 ERIC strategies (% of agreement for > 45% recommendation)	Evidence of ERIC strategy in practice
Outer setting	Systemic conditions	✔			
	External pressure	✔			

Inner setting	Culture	✔	✔	Identify and prepare champions (52%)	‐ Visibility and involvement of leadership ‐ Assigning leadership roles driving implementation
	Access to knowledge and information	✔	✔	Conduct education meetings (79%)	‐ Staff huddles ‐ Preceptor education meetings
				Develop educational materials (59%)	‐ Learning management System e‐module ‐ PowerPoints with quizzes ‐ Posters ‐ Implementation toolkit
				
				
				
				Distribute education material (55%)	‐ Resource binder ‐ Email messages Quality bulletin boards
				
				
	Tension for change	✔			
	Mission alignment	✔			
	Compatibility	✔	✔	Promote adaptability (45%)	‐ RPN in other roles (i.e., circulating nurse)

Innovation characteristics	Relative advantage	✔	✔	Identify and prepare champions (45%)	‐ Surgeon and anesthetist champions
	Adaptability	✔		Promote adaptability (73%)	‐ RPN expanding scope (i.e., second circulating nurse)
	Design	✔	✔	Promote adaptability (48%)	‐ RPN expanding scope (i.e., circulating nurse)

Implementation process	Teaming	✔			
	Assessing context	✔			
	Assessing needs	✔			
	Engaging	✔		Intervene with staff to enhance uptake and adherence (50%)	‐ Team leader workshop ‐ Communities of practice
				Involve staff (59%)	‐ Frontline staff on working groups and implementation committees
				Prepare staff to be active participants (55%)	‐ Focus groups with frontline staff ‐ Change readiness survey
				
	Tailoring strategies	✔			‐ Additional unit orientation ‐ Adapting tools
	Reflecting and evaluating	✔		Audit and provide feedback (56%)	‐ Communities of practice
				Develop and implement tools for quality monitoring (60%)	‐ RPN orientation evaluation survey

Individual characteristics	Motivation	✔		Involve executive boards (45%)	‐ Executive‐level leadership ‐ Executive sponsorship

## 4. Discussion

### 4.1. Implications and Lessons From CFIR–ERIC

This study evaluated the implementation of a new nursing role in an acute care hospital within the main OR, postpartum, and labor and delivery settings, identifying barriers and facilitators to implementation, as well as strategies to support the introduction of the new role in the acute care environment. The data generated mapped to five CFIR domains and 17 constructs, demonstrating a comprehensive process for addressing barriers and amplifying enablers during implementation. Findings suggest that the outer setting (systemic conditions and external pressures) served as an enabler in the implementation process. Many participants mentioned worsened nursing shortages since the pandemic, along with the existing integration of this role in similar institutions. Reports of nursing shortages are not new, even before the pandemic [38]; however, recent reports indicate that COVID‐19’s impact on nurses globally has led to a mass exodus from the profession [39]. In this context, acute care hospitals, aiming to maintain adequate staffing levels for safe, quality patient care, have been identified as engaging in the process of finding optimal nursing models of care [40, 41]. For example, an “advising nurse” role was developed to support novice nurses; however, the number of experienced nurses within the workforce makes sustaining this approach long‐term challenging [40]. Additionally, recent studies examining innovative care delivery models amid ongoing workforce challenges in the UK and the USA found that participants incorporated additional nursing roles across various units as a mitigation strategy to address workforce pressures [41, 42]. Our analysis suggested that the design of the RPN role, as reflected in the scope of practice, depending on the context, led to mixed perceptions. Role clarity in the role facilitated adoption and less ambiguity. Research into the implementation of the Nursing Associates role in England’s acute care system, developed during the COVID‐19 pandemic, reported that clarity around the scope of practice and participants having to quickly adapt and determine how to use the role effectively in practice within a limited time may have caused friction [42, 43].

As this study demonstrated, alternative, sustainable nursing models are critical to address the chronic issues with the nursing workforce; yet, the implementation context also influences the introduction of such models. For example, within the inner setting, it was found that the organizational culture informed by being historically an all‐RN nursing care model with limited exposure to the RPN role may have impacted implementation efforts. Similarly, other studies have cited ongoing challenges around nurses adapting to new models, highlighting a desire to work according to previous models despite lower nurse satisfaction [44, 45]. Nurse leaders also echoed this finding, noting initial challenges with the introduction of another nursing role, as staff adjusted to the change in practice and scope [41, 42]. Yet, this study showed that, despite the organizational climate, the chronic workforce shortage, the alignment of the organizational mission, and the compatibility of the role within the existing hospital system and processes were considered determinants of successful implementation. Therefore, identifying these drivers and other CFIR inner setting constructs is an important consideration of the context when introducing changes in the clinical environment. These findings underscore the active role of the inner setting, or the context, within the implementation process of an innovation [46].

Calls are made to tailor implementation processes and strategies to facilitate the fit of context and innovation [47]. This study demonstrated evidence of efforts to customize strategies to enhance implementation enablers and reduce barriers. Using the ERIC matching tool, these implementation strategies addressed barriers or enhanced efforts, for example, identifying champions, incorporating an audit and feedback loop, and preparing staff to participate actively, which were identified for future scale and spread of the role. The implementation strategies identified through the ERIC mapping aligned with several determinants identified in the CFIR analysis, particularly those related to stakeholder engagement, leadership support, and workforce planning. However, additional strategies, such as structured processes, targeted education for existing staff, and ongoing feedback mechanisms, may further address barriers to role understanding and team integration. According to other research, adapting strategies increases the likelihood of successful implementation by targeting specific organizational needs, fitting them to their unique environment, and directing resources more efficiently and effectively [48, 49].

Finally, the implementation process captured a significant amount of narrative on several CFIR constructs, including teaming, assessing context and needs, and reflecting and evaluating. Overall, the process consistently considered patients’ and clinicians’ needs; provided education, information, and engagement opportunities to address concerns or support learning; and created mechanisms for reflection to evaluate the implementation process, for example, through the CoPs. Notably, this level of commitment to the implementation process has been previously identified in studies as a facilitator [50]. For example, CoPs effectively support sensemaking by sharing information and experiences between implementation team members and, in this case, the innovation, RPNs [51, 52].

Overall, findings suggest that the introduction of the RPN role was partially successful, with several facilitators supporting implementation, including leadership engagement, organizational readiness, and perceived workforce benefits. However, challenges related to role clarity, understanding of the scope of practice, and team integration remained evident. These findings suggest that while the implementation strategies used supported early adoption of the role, additional strategies focused on role clarification, interprofessional communication, and ongoing education may further strengthen integration of the RPN role within acute care settings.

### 4.2. Strengths and Limitations of the Study

This study has several strengths worth noting. To our knowledge, it is the first to apply the CFIR and ERIC frameworks in evaluating the implementation of the RPN role. Using these widely recognized frameworks to analyze the data by influential factors helped explain the results and increased the rigor of the findings. While the CFIR framework provides a structured approach applicable across healthcare contexts, the specific determinants identified in this study reflect the organizational and workforce context in which the RPN role was introduced. These findings may therefore provide insights to inform similar workforce implementation efforts, while recognizing that implementation processes remain context‐dependent. This study also has several limitations. First, it was conducted within a single acute care hospital, which may limit the transferability of findings to other healthcare settings with different organizational structures or workforce models. Second, because the findings are based on self‐reported experiences, there may be some social desirability response bias. This was mitigated through strategies such as emphasizing confidentiality and asking follow‐up questions to explore responses that addressed this. Third, interviews were conducted between three and 8 months following role introduction and therefore reflect early implementation experiences. While some participants described iterative adaptations (e.g., refinements to orientation and role clarity), key barriers and facilitators, such as role clarity, access to knowledge, and team integration, were consistently reported across participants. Future longitudinal research may be needed to examine how these experiences evolve over time.

### 4.3. Recommendations for Future Research

According to the implementation literature, the CFIR framework can be considered an explanatory framework [53]. Therefore, future research could consider identifying the implementation process, including the strategies, with evaluation outcomes mapped to a consistent taxonomy of implementation indicators [54]. This would support administrators and academics within health systems in generating implementation science data, thus contributing scientifically to using evidence‐based approaches to implementation processes. Given how complex and critical leadership is to introducing change [55], there is also an opportunity to explore how hospital leadership influences implementation and related processes. Therefore, taking a more in‐depth understanding and investigation that includes leadership’s influence and breadth in future research could enhance future improvement efforts. Finally, the sustainability of CoPs supporting the integration of new staff is another area of exploration, given the retention issues many hospitals face.

### 4.4. Conclusion

Using an implementation science lens, this study identified key barriers and facilitators influencing the introduction of the RPN role within an acute care nursing skill mix model. Facilitators included leadership engagement, organizational readiness, and perceived workforce benefits, while barriers primarily related to role clarity, understanding of the scope of practice, and integration within existing nursing teams. Applying the CFIR–ERIC approach enabled the identification of implementation determinants and strategies that supported the introduction of the role. These findings highlight the importance of clear role definition, targeted education, and ongoing communication to support the successful integration of new nursing roles in acute care settings. Future implementation efforts should consider these factors when introducing workforce innovations to address nursing shortages.

## Funding

This study was supported by the Registered Practical Nurses Association of Ontario (WeRPN) Research Grant and the Science of Care, Centre for Nursing Excellence at Sinai Health.

## Disclosure

The funder had no role in the design of the study; in the collection, analysis, or interpretation of data; or in the writing of the manuscript.

## Conflicts of Interest

The authors declare no conflicts of interest.

## Supporting Information

Additional supporting information can be found online in the Supporting Information section.

## Supporting information


**Supporting Information 1** Supporting Information 1. COREQ Checklist Table 1.

**Supporting Information 2** Supporting Information 2. Supporting Information Table 2: The CFIR and ERIC domains with corresponding short descriptions.

**Supporting Information 3** Supporting Information 3. Supporting Information Table 3: The interview guides used to support the interview process.

## Data Availability

The data that support the findings of this study are not publicly available due to ethical compliance requirements and institutional research ethics restrictions but may be available from the corresponding author upon reasonable request and with appropriate ethical approvals.
