# Supplementary material for: Exploring the Implementation of a New Nursing Role Using the CFIR–ERIC Approach: A Qualitative Study
Source: J Nurs Manag. 2026 May 16;2026:8513187. doi: 10.1155/jonm/8513187 (PMC13179512; doi:10.1155/jonm/8513187)
Supplement: Supplementary file 3 — Supporting Information 3 Supporting Information 3. Supporting Information Table 3: The interview guides used to support the interview process. [file JONM-2026-8513187-s003.docx]

Welcome to the interview session. Thank you for taking the time to participate. Your insights are invaluable to our research. Before we begin, I want to emphasize the importance of confidentiality. Your participation and responses will be treated with the utmost respect and privacy. Any information shared during this interview will only be used for research purposes and will not be disclosed to anyone outside the research team. Please let us know if you have any concerns about confidentiality or other aspects of the interview. We want you to feel comfortable and assured throughout the process. Thank you again for your participation.

1. Demographic questions
   1. What is your current role?
      1. Which unit/department are you based in?
   2. How long have you been in this role?
   3. How long have you been with the organization?
   4. How many years of experience do you have?

| CFIR Domain | Semi-structured interview questions:  **Organizational leaders/facilitators (e.g., Senior executives, directors)** |
| --- | --- |
| Outer setting | 1. What might have been some of the reasons for introducing RPNs at MSH? 2. How will the RPN role meet the needs of patients and Sinai Health? In what ways? |
| Inner setting | 1. How would you describe the culture of your organization? Of your own setting or unit? 2. How do you think Sinai Health’s culture (general beliefs, values, assumptions that people embrace) has influenced introducing RPNs? 3. What is the general level of receptivity in your organization to introducing RPNs? |
| Intervention Characteristics | 1. What do key partners think of the new role? 2. What is your perception of the quality of the supporting education and training involved in the introduction of RPNs?    1. Can you share more specifically to the RPN hires?    2. Can you share more specifically to other staff about the introduction of RPNs? |
| Characteristics of Individuals | 1. How familiar are you with the RPN role? 2. What do you know about the implementation? 3. Do you think the RPN role will be effective in your setting? 4. How involved were you/What has your role been introducing the RPNs into the nursing skill mis? How involved were you? |
| Process | 1. What have you done (or what do you plan to do) to get a plan in place to implement the RPN role? 2. Can you describe the plan for implementing the intervention? 3. What might be other key barriers or facilitators to the introduction of RPNs? |

| CFIR Domain | Semi-structured interview questions:  **Implementation leaders (e.g., nurse educators, patient care managers (PCMs)** |
| --- | --- |
| Intervention Characteristics | 1. How familiar are you with the RPN role? 2. Thinking about the introduction of RPNs at MSH, what might be some advantages of having RPNs as part of the nursing skill mix? 3. What might be some disadvantages of having RPNs as part of the nursing skill mix? 4. How well has the RPN role been received at MSH? |
| Outer setting | 1. How well does the new role meet the needs of patients and the organizational needs and resources, and in what ways? |
| Inner setting | 1. What supports were available to help you to plan for the introduction of RPNs? 2. How do you think Sinai Health’s culture (general beliefs, values, assumptions that people embrace) has influenced introducing RPNs? |
| Characteristics of Individuals | 1. What has been your motivation to help ensure the implementation is successful? 2. How involved were you/What has your role been introducing the RPNs into the nursing skill mis? How involved were you? |
| Process | 1. What strategies were put in place/changes made to ensure successful implementation of the RPN role? 2. ~~What kind of strategies did you use or develop as you worked on introducing RPNs at MSH?~~ 3. What might be other key barriers or facilitators to the introduction of RPNs? |

| CFIR Domain | Semi-structured interview questions:  **Nursing staff involved in providing care (e.g., RPNs, RNs**) |
| --- | --- |
| Intervention Characteristics | 1. How familiar are you with the RPN role? 2. What might be some advantages of having RPNs as part of the nursing skill mix? 3. What might be some disadvantages of having RPNs as part of the nursing skill mix? 4. How well has the RPN role been received at MSH? 5. What might be some challenges with the new role? |
| Outer setting | 1. How well does the new role meet the needs of patients and the organizational needs and resources, and in what ways? |
| Inner setting | 1. What supports were available to help you to plan for the introduction of RPNs? 2. How do you think Sinai Health’s culture (general beliefs, values, assumptions that people embrace) has influenced introducing RPNs? 3. How were you involved in the plan for introducing RPNs? |
| Characteristics of Individuals | 1. What has been your motivation for helping to ensure the implementation is successful? 2. How confident are you in the organization being able to spread the RPN role into other practice settings? about being able to sustain the role? |
| Process | 1. What kind of strategies did you use or develop as you worked on introducing RPNs at MSH? 2. What might be other key barriers or facilitators to the introduction of RPNs? |
